# The Unexpected Guest: Basal Cell Carcinoma on a Covered Site

**DOI:** 10.7759/cureus.78259

**Published:** 2025-01-30

**Authors:** Ulka Pandurangi, R Dharani, Leena Dennis Joseph, Anuradha Priyadarshini, Adikrishnan Swaminathan

**Affiliations:** 1 Dermatology, Sri Ramachandra Institute of Higher Education and Research, Chennai, IND; 2 Pathology, Sri Ramachandra Institute of Higher Education and Research, Chennai, IND

**Keywords:** basal cell carcinoma, hedgehog signalling, nonmelanoma skin cancer, non-sun exposed area, skin cancer

## Abstract

Basal cell carcinoma (BCC) is the most prevalent form of skin cancer; it is slow-growing and locally invasive with an extremely low likelihood of metastasis. The activation of the Hedgehog (HH) signaling pathway is implicated in nearly all cases, with ultraviolet radiation being the primary risk factor. While BCC typically occurs in areas of chronic sun exposure, it can rarely develop in non-sun-exposed areas, as demonstrated by our case of a 68-year-old Indian woman presenting with a BCC lesion on her left hip. Dermoscopy revealed features suggestive of basal cell carcinoma which was subsequently confirmed as adenoid type on histopathological examination. The patient underwent a successful wide excision of the lesion with clear margins, and post-operative healing was satisfactory. This case highlights the rare occurrence of BCC in non-sun-exposed areas and underscores the importance of early diagnosis and treatment. Regular follow-up is essential, as patients with a history of BCC are at high risk for subsequent skin cancers.

## Introduction

Basal cell carcinoma (BCC) is the most prevalent type of skin cancer constituting 80% of all non-melanoma skin cancers with its incidence exhibiting a twofold increase between the ages of 40 and 70 [1,2]. Activation of the Hedgehog (HH) signalling pathway has been implicated in almost all cases. Ultraviolet radiation from sun rays is the most important risk factor. Other contributing factors include ionizing radiation, arsenic exposure, immunosuppression, and genetic predisposition [3]. It develops in areas with chronic sun exposure such as head and neck in 85% of cases and rarely in covered areas [4]. We herein report a case of BCC developing over the hips in an Indian woman.

## Case presentation

**Figure 1:**
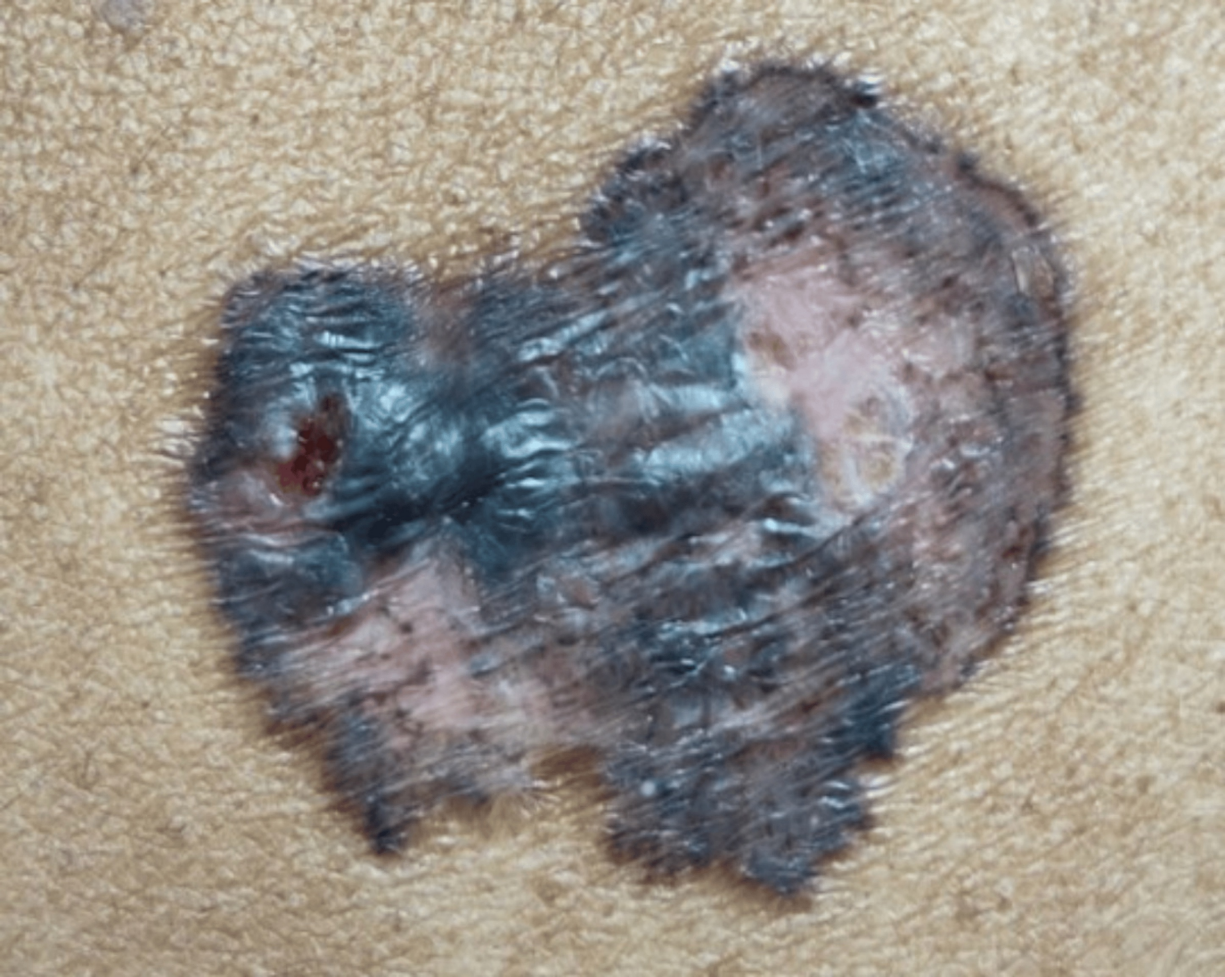
Well-defined, hyperpigmented plaque measuring 4 cm X 3 cm with 2 mm area of erosion

A 68-year-old woman, of Fitzpatrick skin type 4, presented with a dark skin lesion on her left hip that was insidious in onset and gradually increased in size over 20 years. It was associated with pain and itching for two weeks. There was no history of trauma, injury or radiation exposure at the site. She was post-menopausal with no comorbidities and no significant past medical, surgical, or family history. General and systemic examination was normal. A single 4 x 3 cm well-defined, non-tender, hyperpigmented thin plaque with 2 mm erosion on the surface was present over the left hip (Figure 1).

**Figure 2:**
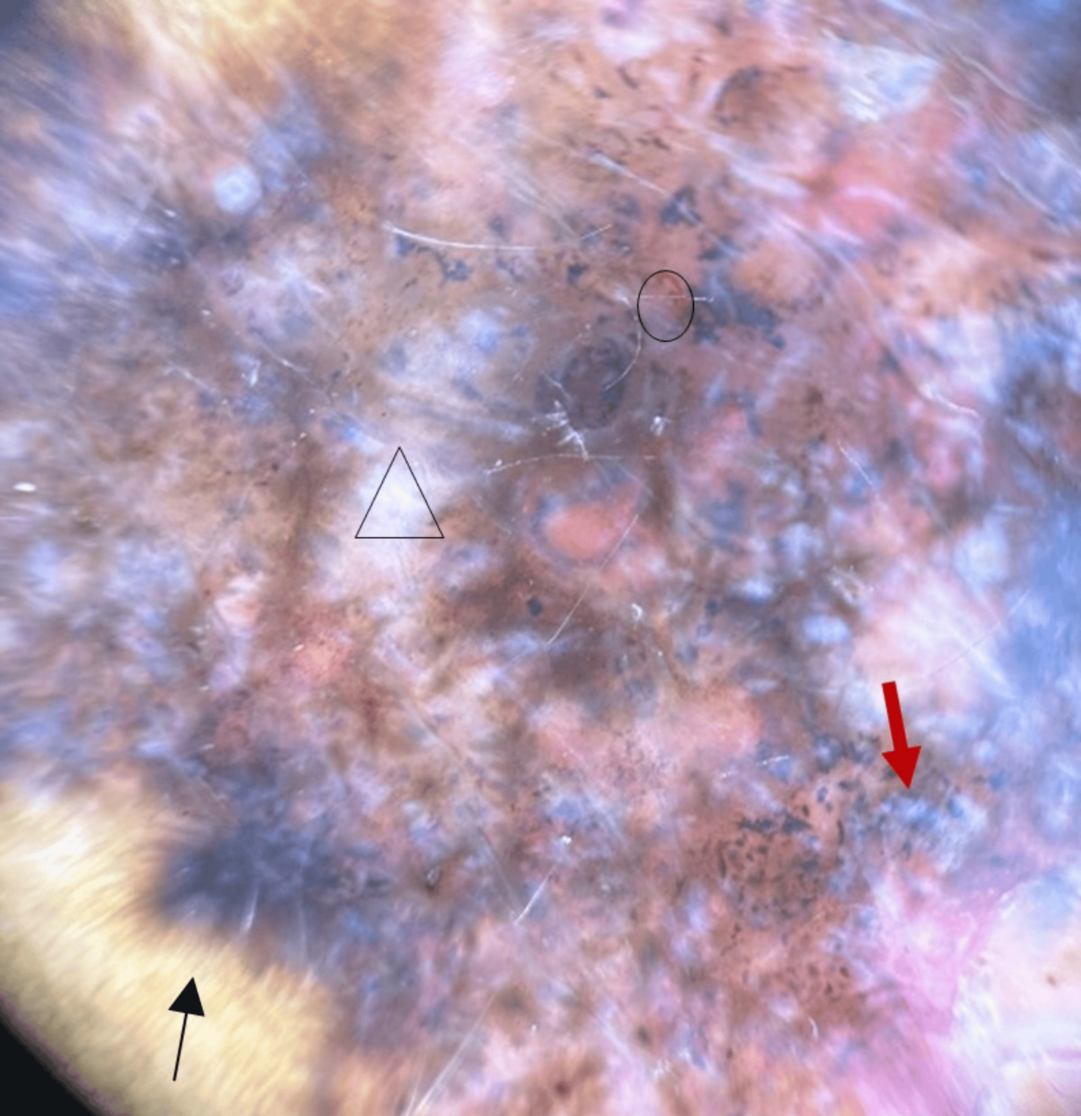
Dermoscopic examination showing multiple blue-grey nests and globules (red arrow), areas of white clods (triangle), superficial fine telangiectasia (circle), and peripheral leaf-like areas (black arrow)

There was no regional lymphadenopathy. A dermoscopic examination of the lesion showed multiple blue-grey nests and globules, areas of white clods, superficial fine telangiectasia, and peripheral leaf-like areas suggestive of BCC (Figure 2).

**Figure 3:**
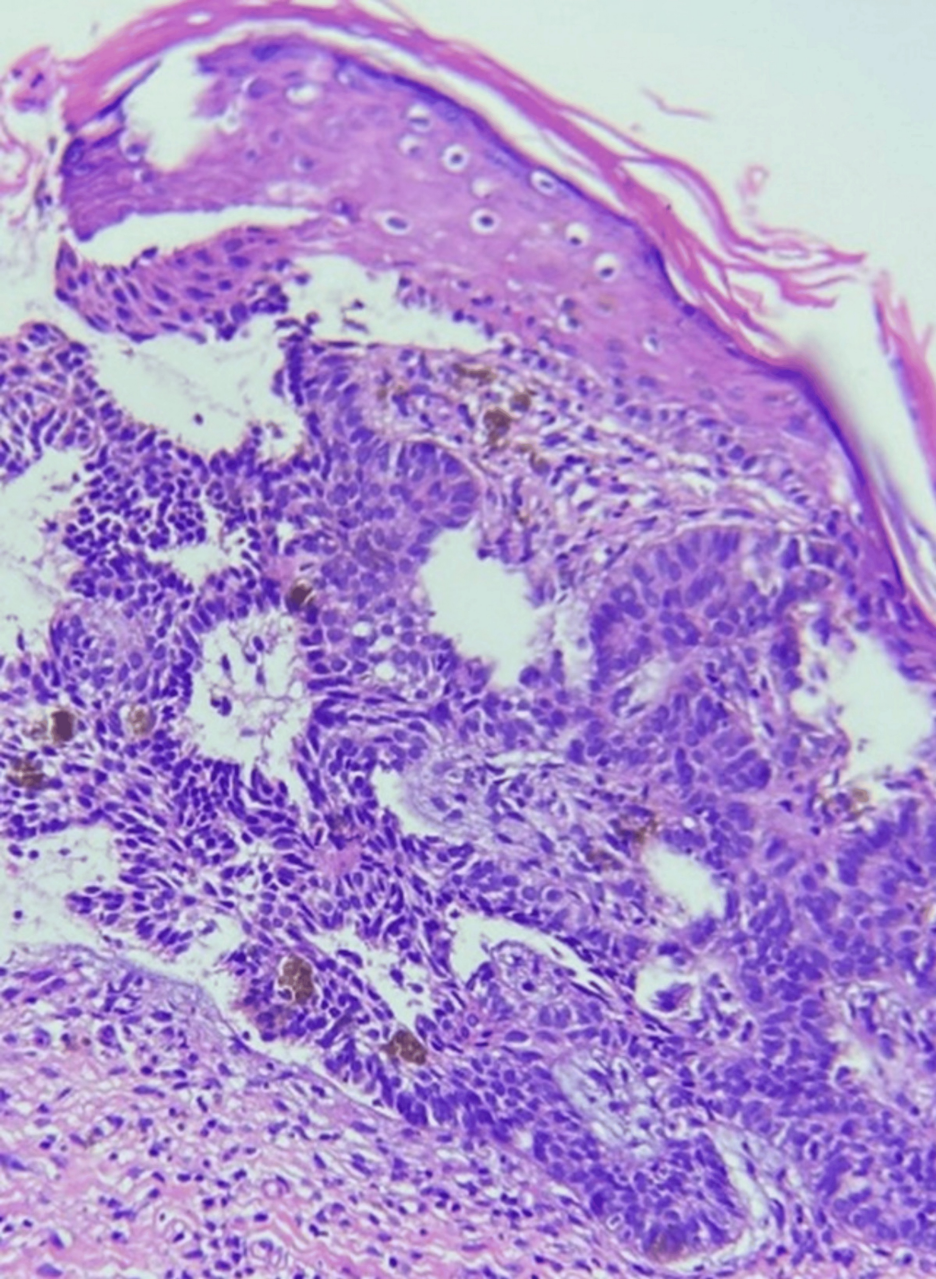
Histopathological examination of skin punch biopsy showed palisading basaloid cells extending into the dermis with areas of retraction clefts. A dense lymphocytic infiltrate, intermixed with pigment-laden macrophages seen in the upper dermis. (hematoxylin and eosin staining, 200x view)

A 4 mm skin punch biopsy was taken, which on histopathological examination showed palisading basaloid cells extending into the dermis with areas of retraction clefts. A dense lymphocytic infiltrate, intermixed with pigment-laden macrophages, was observed in the upper dermis. This confirmed the diagnosis of BCC (Figure 3).

**Figure 4:**
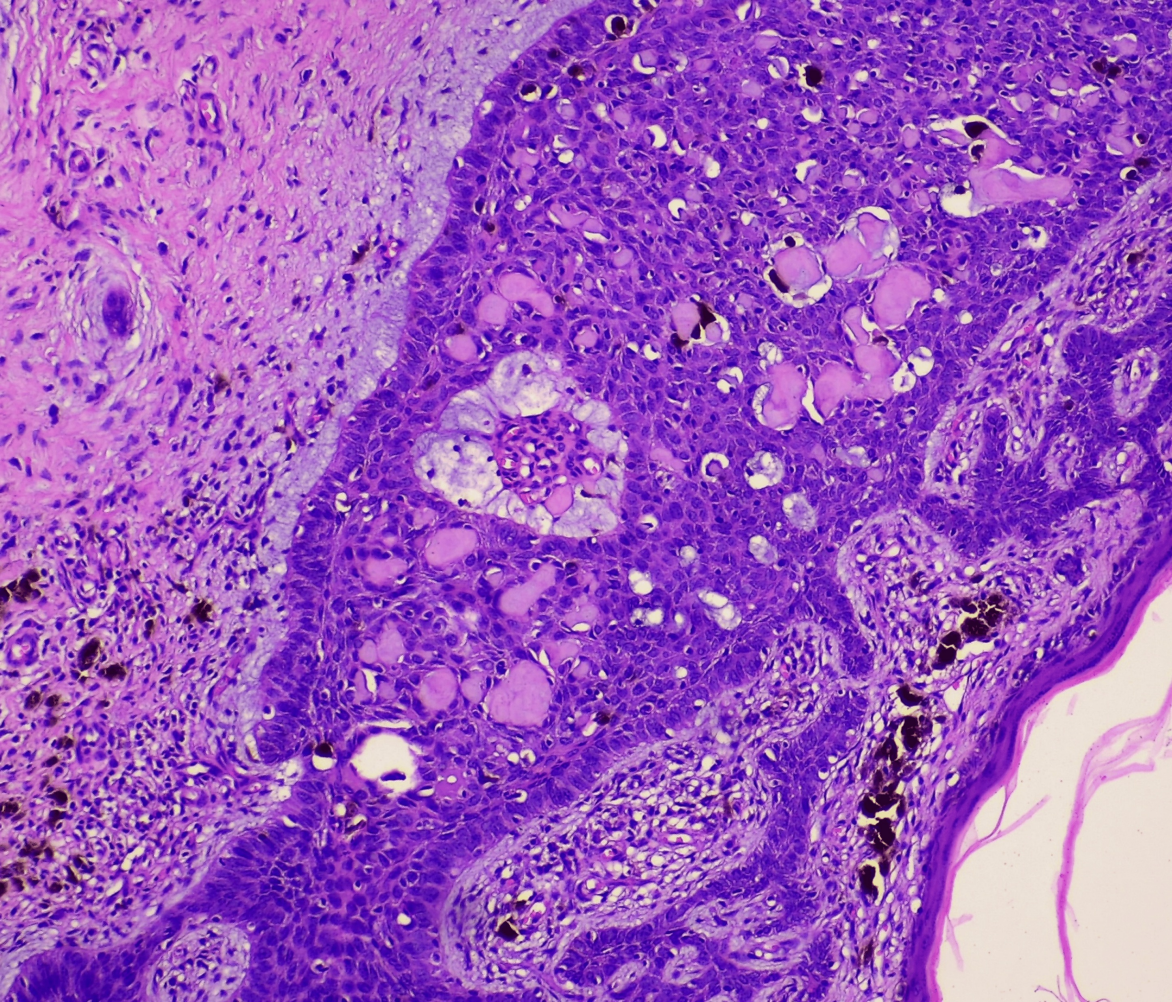
Histopathological examination of excised specimen showing tumour arising from basal layer with prominent reticulate pseudoglandular pattern surrounded by mucinous stroma, peripheral palisading cells (hematoxylin and eosin staining, 20x view)

The patient underwent wide excision of the lesion with a 15 mm margin. Histopathological examination of the excised specimen showed tumour arising from the basal layer with a prominent reticulate pseudoglandular pattern surrounded by mucinous stroma and presence of peripheral palisading cells further confirming the diagnosis to be BCC, adenoid type (Figure 4).

All margins were found to be clear of the tumor with no lymphovascular/perineural invasion. At three weeks postoperatively, the site of excision showed good healing. The patient was advised regular follow-ups.

## Discussion

Only up to 15% of BCC cases occur on non-sun-exposed areas of the body [4]. The lesion in our case was present on the hip, which is a non-sun-exposed area in our patient. The activation of the HH pathway is implicated in nearly all cases, with ultraviolet radiation being the primary risk factor. Some of the contributing factors to the development of BCC in non-sun-exposed areas include the use of tanning beds, radiation exposure, human papillomavirus infection, arsenic in diet, and immune suppression.

BCC, similar to squamous cell carcinoma, can also develop in areas with scars, draining sinuses, ulcers, burn sites, and chronic inflammation [5]. In areas of trauma, the persistent release of inflammatory mediators, growth factors, or other chemical signals is said to contribute to the initiation of BCC carcinogenesis [6]. Carr AV et al. presented a case of BCC in the perianal region highlighting chronic irritation and areas of scar to be at higher risk [1].

Certain inherited mutations cause increased genetic susceptibility to BCC. Gorlin syndrome, also known as nevoid basal cell carcinoma syndrome, arises from germline mutations in the PTCH1 gene, a key component of the Hedgehog signaling pathway leading to early onset BCC. Another example is Bazex-Dupré-Christol syndrome, caused by mutations in ACTRT1, which similarly affects the HH pathway. Inherited deficiencies in DNA repair pathways such as in xeroderma pigmentosum also lead to increased UV-induced DNA damage [7,8].

The present case did not have any apparent risk factor. Similarly, cases of BCC presented by Saravanakumar A et al. [4] over the thighs, by Voris Tand et al. [9] over the back and by Varadarajan VV et al. [10] in the oral cavity also did not have any clear risk factor. Research by Martinez-Ortega et al. [11] aimed at understanding BCC oncogenesis in atypical locations. Their study highlights key factors such as embryological gene expression which may predispose specific regions to malignancy, touch dome cell involvement suggesting specialized mechanosensory cells as potential origins for BCC in unique areas and non-canonical Hedgehog pathway activation, which may play a role in driving tumorigenesis in distinct microenvironments.

Dermoscopy has greatly enhanced the detection of atypical BCCs, especially in non-sun-exposed areas where clinical suspicion is lower like that of our case. A study by Lallas et al. demonstrated that dermoscopy reveals subtle pigmentation undetectable to the naked eye, improving diagnostic sensitivity and specificity. Key dermoscopic features, such as arborizing vessels, blue-gray ovoid nests, and maple leaf-like areas, facilitate early recognition, even in cases lacking classic clinical signs [12].

BCC can be classified clinically or histologically. Seven clinical types are recognized: nodular (commonest), superficial, pigmented, infiltrative, morpheaform, fibroepithelial and infundibulocystic. Histologically they can be classified as superficial, nodular, sclerosing, pigmented, basosquamous, with adnexal differentiation, and fibroepithelial. Nodular BCC is further classified into nodulocystic, keratotic, and adenoid types [13]. Compared to other common subtypes, adenoid BCC is regarded as a low-grade malignancy [14,15].

Lesions can be classified as low risk or high risk based on clinical, pathological and margin involvement as per British guidelines [16]. Low-risk BCCs are primary lesions that occur in non-immunosuppressed patients with no history of prior radiotherapy. Pathologically, these lesions are nodular or superficial without basosquamous differentiation, and the level of invasion is limited to the dermis and subcutaneous fat, with well-defined borders. The maximum clinical diameter is ≤ 20 mm in the trunk and extremities, excluding the genitals, nail units, hands, pretibial regions, ankles, and feet, and ≤ 10 mm in the scalp, forehead, cheeks, and neck. Additional criteria include a depth of invasion less than 6 mm, no evidence of perineural invasion, a pathological TNM stage of pT1 (Union for International Cancer Control (UICC) Staging system) (maximum diameter ≤ 20 mm), and histological margins free of tumor involvement. Excision is the most common treatment modality, with peripheral margins up to 15 mm for high-risk tumors and from 2 to 5 mm for low-risk tumors [16].

Other treatment options for basal cell carcinoma include cryosurgery, topical 5-fluorouracil or imiquimod, electrodesiccation and curettage, intralesional injection of interferon alpha or bleomycin, and photodynamic therapy. In cases of advanced or locally aggressive disease, systemic therapy options such as vismodegib, a hedgehog pathway inhibitor, may be considered [8]. Recent studies have explored the use of anti-PD-1 monotherapy in advanced or metastatic BCC [17]. Patients with a history of BCC are at a high risk of developing subsequent skin cancers of various types, making regular follow-up essential [18].

## Conclusions

Basal cell carcinoma has various morphological forms and sites of presentation. This case report of a basal cell carcinoma over the hip in an Indian woman highlights the importance of considering BCC as a potential diagnosis even in atypical and non-sun-exposed areas across all patient populations. The occurrence of BCC in such locations is rare and poorly understood. Despite the absence of identifiable risk factors, the lesion was diagnosed as adenoid-type BCC, a low-grade malignancy. Regular follow-up is crucial for early detection and management of any subsequent skin malignancies, given the increased risk of recurrence and new skin cancers in individuals with a history of BCC. This case adds to the limited body of knowledge regarding BCC in non-sun-exposed areas and underscores the importance of individualized patient care and continuous monitoring. It also highlights importance of dermoscopy as a valuable tool to avoid misdiagnosis, especially at atypical sites.

## References

[1] Carr AV, Feller E, Zakka FR, Griffith RC, Schechter S (2018). A case report of basal cell carcinoma in a non-sun-exposed area: a rare presentation mimicking recurrent perianal abscess. Case Rep Surg.

[2] Gangan R (2022). Basal cell carcinoma: epidemiology. J Skin Sex Transm Dis.

[3] Cameron MC, Lee E, Hibler BP (2019). Basal cell carcinoma: epidemiology; pathophysiology; clinical and histological subtypes; and disease associations. J Am Acad Dermatol.

[4] Saravanakumar A, Venkatesh A, Aziz AA, Anand S, Sreedharan AV, Jawalgi AP (2023). Basal cell carcinoma in a non-sun exposed site: a rare case report. IP Indian J Clin Exp Dermatol.

[5] Crowson AN (2006). Basal cell carcinoma: biology, morphology and clinical implications. Mod Pathol.

[6] Noodleman FR, Pollack SV (1986). Trauma as a possible etiologic factor in basal cell carcinoma. J Dermatol Surg Oncol.

[7] Kilgour JM, Jia JL, Sarin KY (2021). Review of the molecular genetics of basal cell carcinoma; inherited susceptibility, somatic mutations, and targeted therapeutics. Cancers (Basel).

[8] Marzuka AG, Book SE (2015). Basal cell carcinoma: pathogenesis, epidemiology, clinical features, diagnosis, histopathology, and management. Yale J Biol Med.

[9] Voris T, Quinn R, Elahi M, Alani H (2003). Subcutaneous basal cell carcinoma: a case report. Can J Plast Surg.

[10] Varadarajan VV, Nasri E, Dziegielewski PT (2020). Basal cell carcinoma of the oral cavity: a case report. Otolaryngol Case Rep.

[11] Martinez-Ortega JI, Perez Hernandez FJ, Reyes Garcia LN (2023). Basal cell carcinoma in the periungual region: a rare case and pathogenesis insights. Cureus.

[12] Lallas A, Argenziano G, Kyrgidis A (2014). Dermoscopy uncovers clinically undetectable pigmentation in basal cell carcinoma. Br J Dermatol.

[13] Balakrishnan S (2022). Basal cell carcinoma - pathology. J Skin Sex Transm Dis.

[14] Sethi N, Sharma A, Singh C, Pandia K, Gupta A (2021). Adenoid basal cell carcinoma:a rare variant and a diagnostic dilemma. Clin Dermatol Rev.

[15] Tambe SA, Ghate SS, Jerajani HR (2013). Adenoid type of basal cell carcinoma: rare histopathological variant at an unusual location. Indian J Dermatol.

[16] Nasr I, McGrath EJ, Harwood CA (2021). British Association of Dermatologists guidelines for the management of adults with basal cell carcinoma 2021. Br J Dermatol.

[17] In GK, Nallagangula A, Choi JS (2022). Clinical activity of PD-1 inhibition in the treatment of locally advanced or metastatic basal cell carcinoma. J Immunother Cancer.

[18] Duarte AF, Sousa-Pinto B, Haneke E, Correia O (2018). Risk factors for development of new skin neoplasms in patients with past history of skin cancer: a survival analysis. Sci Rep.

